# Small non-coding RNA transcriptomic profiling in adult and fetal human brain

**DOI:** 10.1038/s41597-024-03604-6

**Published:** 2024-07-12

**Authors:** Marharyta Smal, Domenico Memoli, Elena Alexandrova, Domenico Di Rosa, Ylenia D’Agostino, Fabio Russo, Giorgio Giurato, Giovanni Nassa, Roberta Tarallo, Alessandro Weisz, Francesca Rizzo

**Affiliations:** 1 Laboratory of Molecular Medicine and Genomics, Department of Medicine, Surgery and Dentistry ‘Scuola Medica Salernitana’, University of Salerno, 84081 Baronissi, SA Italy; 2 Medical Genomics Program, AOU ‘S. Giovanni di Dio e Ruggi d’Aragona’, University of Salerno, Salerno, Italy; 3 Genome Research Center for Health - CRGS, Campus of Medicine - University of Salerno, 84081 Baronissi, SA Italy

**Keywords:** Piwi RNAs, miRNAs, Small RNAs

## Abstract

Small non-coding RNAs (sncRNAs) make up ~1% of the transcriptome; nevertheless, they play significant roles in regulating cellular processes. Given the complexity of the central nervous system, sncRNAs likely hold particular importance in the human brain. In this study, we provide sncRNA transcriptomic profiles in a range of adult and prenatal brain regions, with a focus on piRNAs, due to their underexplored expression in somatic cells and tissue-specific nature. Using the WIND workflow, which combines two detection methods, we found 1333 (731 miRNAs, 249 piRNAs, 285 snoRNAs, and 68 other sncRNAs) and 1445 unique sncRNAs (770 miRNAs, 307 piRNAs, 289 snoRNAs, and 79 other sncRNAs) in developing and adult brains, respectively. Significant variations were found upon comparison of fetal and adult brain groups, with 82 miRNAs, 17 piRNAs, and 70 snoRNAs enriched in fetal brains and 22 miRNAs, 11 piRNAs in adult brains. This dataset represents a valuable resource for exploring the sncRNA roles in brain function, their involvement in neurological diseases, and the molecular mechanisms behind brain region interactions.

## Background & Summary

Small non-coding RNAs (sncRNAs) constitute a large family of endogenously expressed RNA transcripts, less than 200 nucleotides in size, that are not translated into proteins and compose less than 1% of the human transcriptome^1,2^. These RNAs originate from numerous distinct biogenesis pathways and play a pivotal role in regulating cellular functions. SncRNAs encompass various family members that include microRNAs (miRNAs), small interfering RNAs (siRNAs), small nuclear RNAs (snRNAs), small nucleolar RNAs (snoRNAs), and piwi-interacting RNAs (piRNAs). Although the exact function of many sncRNAs remains unknown, multiple studies have revealed their involvement in regulating mRNA transcription, alternative splicing, translation, post-translational regulation, and epigenetic modifications such as chromatin structure and RNA editing, as well as in DNA damage response^3–5^.

Small non-coding RNAs are pertinent across all mammalian cell types but are likely of particular importance in the brain, which contains the highest number of unique transcripts not present in other tissues^6–8^. The human brain is a complex organ composed of many different functional regions. The orchestration of activities within these regions and their interplay could potentially be modulated by sncRNAs. Within the brain, a substantial number of sncRNAs exhibit abundant expression, displaying specificity for brain regions and cell types^9,10^. Studies have indicated that alterations in sncRNA expression are linked to pathological processes in the brain, thereby contributing to the development of psychiatric and neurodegenerative disorders^3,11–13^.

In the context of the brain, miRNAs remain the most extensively studied non-coding RNA species. It has been reported that approximately 70% of all miRNAs are expressed there^14^, which is unsurprising, considering the cellular and transcriptional complexity of the central nervous system. MicroRNAs, known as negative regulators of gene expression, have been linked to learning and memory function^15^ as well as implicated in the pathogenesis of neurodegenerative diseases, including Alzheimer’s disease^16,17^.

Other sncRNAs that may potentially affect brain function but have been less investigated include snRNAs, snoRNAs, and piRNAs. SnRNAs are the components of the spliceosome that may regulate alternative splicing and cause severe brain abnormalities when mutated^18^. SnoRNAs are primarily involved in the post-transcriptional modification of other RNA species and were shown to be involved in the development of Prader-Willi syndrome and presumably schizophrenia^19,20^. PiRNAs, together with Piwi-class Argonaute proteins, form a silencing complex capable of suppressing transposable elements and thereby maintaining genome integrity^21^. Initially discovered and found to be most abundant in germline cells, piRNAs have also been identified in somatic tissues, including the brain^22,23^. In a study in Aplysia, it was found that the Piwi/piRNA complex promotes serotonin-dependent methylation in the *CREB2* promoter, leading to enhanced long-term synaptic facilitation and memory storage^24^. In Mili/piRNA-deficient (Mili−/−) mice, brain genomic DNA showed preferential hypomethylation within intergenic and LINE1 promoter areas. Furthermore, mutant mice exhibited behavioral deficits, including hyperactivity and reduced anxiety^25^. However, when it comes to the human brain, apart from a few pilot studies on the association of piRNAs with Alzheimer’s^26,27^, Huntington’s^28^, Parkinson’s^29^ and other neurodegenerative diseases^30^, there is a lack of data on their expression.

Given the scarcity of data on piRNA expression in the human brain and the challenges arising from their poor conservation among species and tissue-specific nature^31^, our study focused on investigating piRNA expression across different brain areas. Although the emphasis in our work is given to piRNAs, a range of other identified sncRNAs is also presented to provide a glimpse into their abundance in the human brain. It is worth noting that despite the availability of tissue-level sncRNA resources, the brain remains significantly underrepresented. To our knowledge, no prior study encompasses a spectrum of expressed small non-coding RNAs in different brain regions from female and male individuals at both embryonic and adult life stages.

**Fig. 1:**
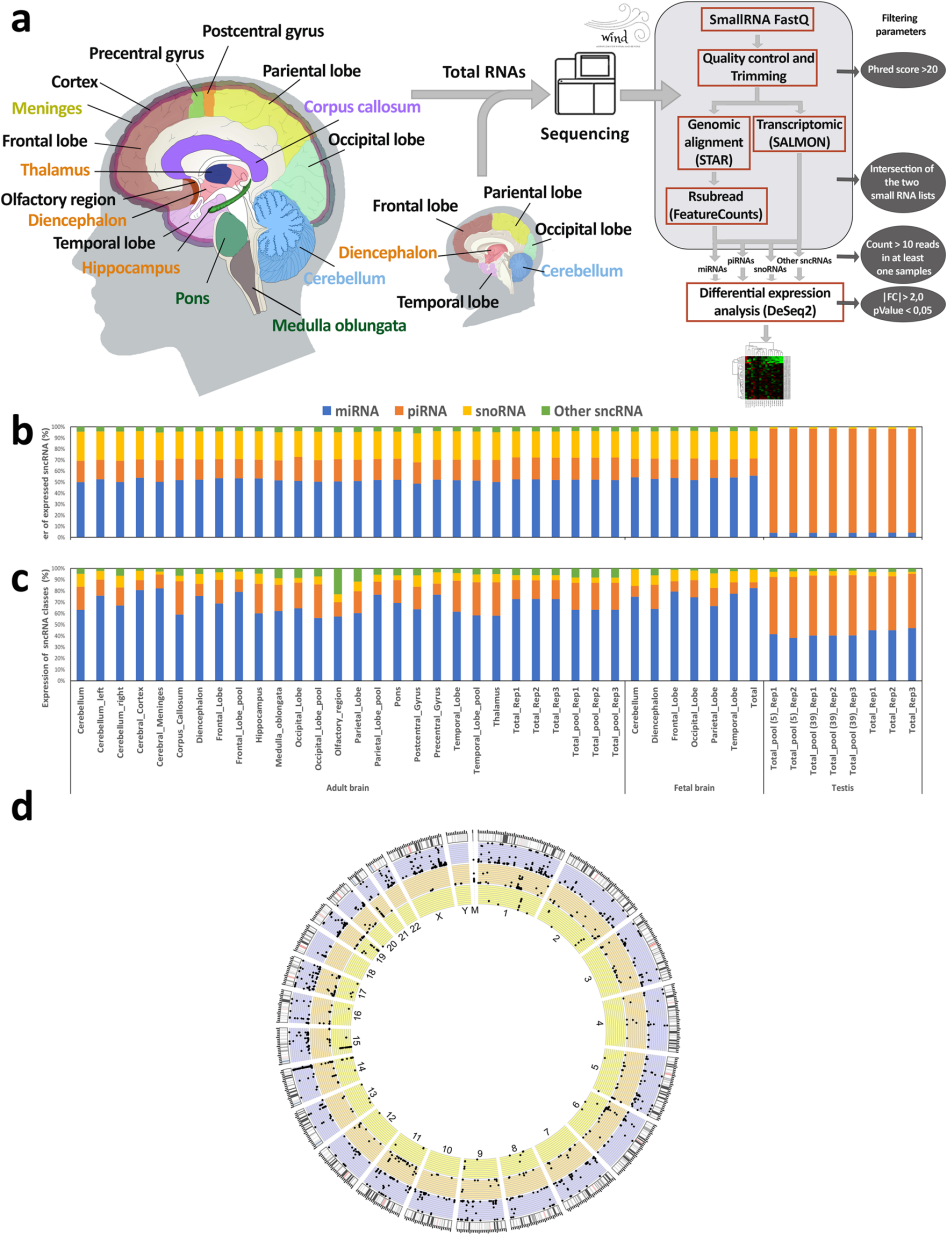
Characterization of small non-coding RNA (sncRNA) expression profiles in the adult and fetal human brain. (**a**) Brain regions subjected to sncRNA transcriptomic profiling and schematic overview of the sncRNA-seq analysis workflow and filtering strategy. The brain macroregions are indicated by different colors: Brain Stem (sea green), Cerebellum (sky blue), Cerebral cortex (black), Corpus Callosum (medium purple), Limbic system (dark orange), Meninges (olive drab). For details see Table 1. (**b**) Abundance of detected sncRNA species per sample, including samples from both the brain and testis. (**c**) Percentage representation of the average expression across all sncRNA populations per sample. (**d**) Circos plot illustrating genomic positions and mean log2 (count + 1) expression levels of miRNAs (blue), piRNAs (orange), and snoRNAs (yellow) detected (reads ≥ 10) in the adult and fetal human brain. Samples that are pools of RNA from different individuals are indicated as pools. M: mitochondrial genome.

Here, we present a dataset containing sncRNA expression profiles across various brain regions (Fig. 1a) from 24 adult and 7 fetal samples (Table 1 and Supplementary Table 1). The profiles were generated using next-generation sequencing and the WIND pipeline^32^, enabling the identification of primary sncRNA species such as miRNAs, piRNAs, snoRNAs, snRNAs, and several other less frequent classes of these RNAs. This bioinformatics workflow was selected since it allows the integration of different databases for piRNA annotation and also implements a dual-method approach to allow a more precise and reliable sncRNA detection and quantification. Since germinal cells exhibit a high variety of piRNAs and are characterized by a high expression of these molecules^33^, we sequenced in parallel 3 samples of human testis RNA, as a control for piRNA and other rare sncRNA detection.

**Table 1:** Sample information.

Sample name	Macroregions	Region	Tissue	Donors	Age status	Sex	Years	Weeks	RIN
Adult_Brain_Cerebellum	Cerebellum	Cerebellum	Brain	Single	Adult	M	27	—	7,2
Adult_Brain_Cerebellum_left	Cerebellum	Cerebellum_left	Brain	Single	Adult	M	29	—	8,4
Adult_Brain_Cerebellum_right	Cerebellum	Cerebellum_right	Brain	Single	Adult	M	82	—	8,7
Adult_Cerebral_Cortex	Cerebral cortex	Cerebral_Cortex	Brain	Single	Adult	M	27	—	6,8
Adult_Cerebral_Meninges	Meninges	Cerebral_Meninges	Brain	Single	Adult	F	82	—	5,9
Adult_Corpus_Callosum	Corpus Callosum	Corpus_Callosum	Brain	Single	Adult	M	26	—	6,7
Adult_Diencephalon	Limbic system	Diencephalon	Brain	Single	Adult	M	26	—	7,7
Adult_Frontal_Lobe	Cerebral cortex	Frontal_Lobe	Brain	Single	Adult	M	29	—	8,2
Adult_Pool_Frontal_Lobe	Cerebral cortex	Frontal_Lobe	Brain	Pool (5)	Adult	M,M,M,M,M	22,26,27,28,29	—	6,2
Adult_Hippocampus	Limbic system	Hippocampus	Brain	Single	Adult	M	27	—	8,2
Adult_Medulla_oblongata	Brain Stem	Medulla_oblongata	Brain	Single	Adult	M	82	—	6,4
Adult_Occipital_Lobe	Cerebral cortex	Occipital_Lobe	Brain	Single	Adult	M	27	—	7,2
Adult_Pool_Occipital_Lobe	Cerebral cortex	Occipital_Lobe	Brain	Pool (5)	Adult	M,M,M,M,M	20,22,23,29,41	—	7,1
Adult_Olfactory_region	Cerebral cortex	Olfactory_region	Brain	Single	Adult	F	87	—	7,4
Adult_Parietal_Lobe	Cerebral cortex	Parietal_Lobe	Brain	Single	Adult	M	49	—	7,3
Adult_Pool_Parietal_Lobe	Cerebral cortex	Parietal_Lobe	Brain	Pool (5)	Adult	M,M,M,M,F	20,24,27,49,61	—	6,8
Adult_Pons	Brain Stem	Pons	Brain	Single	Adult	F	60	—	6,9
Adult_Postcentral_Gyrus	Cerebral cortex	Postcentral_Gyrus	Brain	Single	Adult	M	41	—	5,8
Adult_Precentral_Gyrus	Cerebral cortex	Precentral_Gyrus	Brain	Single	Adult	M	26	—	6,1
Adult_Temporal_Lobe	Cerebral cortex	Temporal_Lobe	Brain	Single	Adult	M	27	—	7,4
Adult_Pool_Temporal_Lobe	Cerebral cortex	Temporal_Lobe	Brain	Pool (5)	Adult	M,M,M,M,M	23,24,26,26,27	—	7,3
Adult_Thalamus	Limbic system	Thalamus	Brain	Single	Adult	M	24	—	6,6
Adult_Total_Rep1		Total	Brain	Single	Adult	M	24	—	8,3
Adult_Total_Rep2									
Adult_Total_Rep3									
Adult_total_pool_Rep1		Total	Brain	Pool (5)	Adult	M,M,M,M,M	21,24,27,27,29	—	8,6
Adult_total_pool_Rep2									
Adult_total_pool_Rep3									
Fetal_Cerebellum	Cerebellum	Cerebellum	Brain	Single	Fetal	F	—	37	7,4
Fetal_Diencephalon	Limbic system	Diencephalon	Brain	Single	Fetal	F	—	28	8,9
Fetal_Frontal_Lobe	Cerebral cortex	Frontal_Lobe	Brain	Single	Fetal	F	—	20	7,6
Fetal_Occipital_Lobe	Cerebral cortex	Occipital_Lobe	Brain	Single	Fetal	M	—	22	7,7
Fetal_Parietal_Lobe	Cerebral cortex	Parietal_Lobe	Brain	Single	Fetal	F	—	24	8,5
Fetal_Temporal_Lobe	Cerebral cortex	Temporal_Lobe	Brain	Single	Fetal	M	—	40	8,9
Fetal_total		Total	Brain	Single	Fetal	F	—	22	6,8
Total_pool (5)_Testis_Rep1		Total	Testis	Single	Adult	M	29	—	7,9
Total_pool (5)_Testis_Rep2									
Total_pool (39)_Testis_Rep1		Total	Testis	Pool (39)	Adult	M	16–64	—	7,8
Total_pool (39)_Testis_Rep2									
Total_pool (39)_Testis_Rep3									
Total_Testis_Rep1		Total	Testis	Pool (5)	Adult	M,M,M,M,M	21,24,24,27,29	—	7,8
Total_Testis_Rep2									
Total_Testis_Rep3									

Each sequenced library yielded between 25.4 and 65.5 million reads mapping to the human genome (Supplementary Table 2). Raw read counts of individual samples were normalized against the total number of mapped reads within the same samples to adjust for differences in library size and sequencing depth. We evaluated the obtained sequencing data using Multidimensional Scaling (MDS) analysis and observed two main clusters corresponding to the brain and testis samples (Supplementary Fig. 1), reflecting significant differences in sncRNA expression between human tissues. Moreover, within the brain sample group, clustering predominantly occurred based on fetal vs adult developmental stages.

Employing a detection threshold of greater than or equal to 10 reads in at least one sample, we identified a total of 1445 unique sncRNAs (770 miRNAs, 307 piRNAs, 289 snoRNAs, and 79 other sncRNAs) in adult brain samples and 1333 (731 miRNAs, 249 piRNAs, 285 snoRNAs, and 68 other sncRNAs) in fetal brain counterparts. In contrast, the testis samples exhibited a high abundance of piRNAs, with 18,441 unique transcripts. Supplementary Table 3 details the number of identified sncRNAs per sample. In various brain areas at both embryonic and adult life stages, miRNA was the type of sncRNA with the highest number of molecules represented in our libraries, followed by snoRNA, piRNA, and other sncRNAs (Fig. 1b). The number of sncRNA transcripts detected in our study is comparable to that in other datasets, which, however, lack information on piRNA molecules^8,34^.

We observed a lower median expression level of the piRNA population in the developing (averaging ∼12%) and adult brain (∼19%) compared to the testis (∼50%), considering all reads assigned to small non-coding RNAs (Fig. 1c). Notably, the highest percentage of piRNA expression (more than 26% of all sncRNAs) was found in adult brain regions like corpus callosum, hippocampus, occipital lobe, temporal lobe, and thalamus, which play crucial roles in receiving and transferring information, memory, and learning^35–38^.

SncRNAs are expressed across all chromosomes in brain tissues (Supplementary Table 4). To illustrate the genomic positions of identified sncRNAs, we employed a circular representation of the human genome (Fig. 1d and Supplementary Fig. 2). Regions enriched with miRNAs, piRNAs, and snoRNAs were found on chromosomes 14, 6, and 15, respectively.

**Fig. 2:**
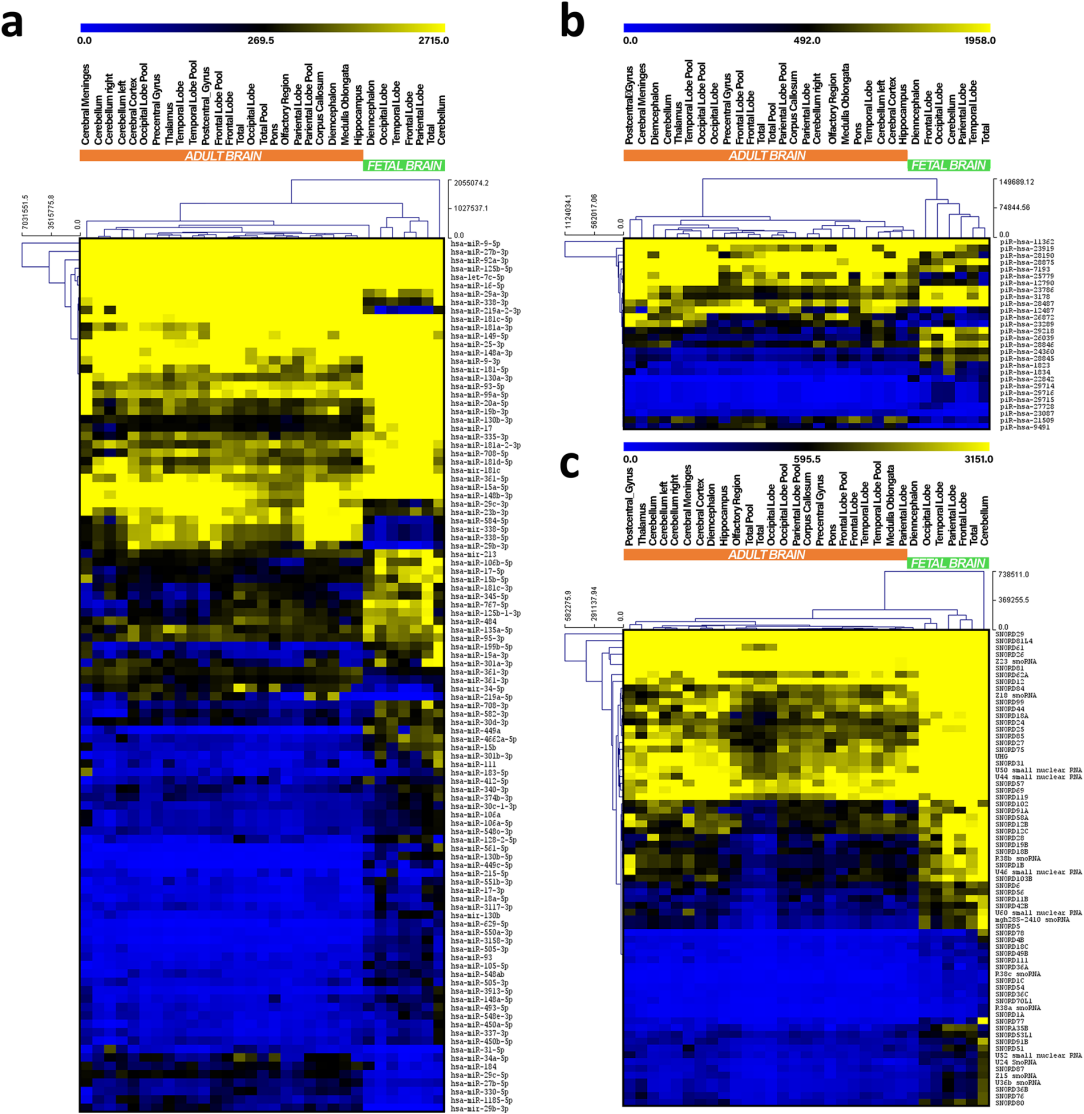
Differential expression analysis. Heatmaps showing differentially expressed miRNAs (**a**), piRNAs (**b**) and snoRNAs (**c**) between adult and fetal brains. Only sncRNAs with a read count ≥ 100 in at least one sample, with |FC| ≥ 2.0, and adjusted p-value ≤ 0.05 were considered. Expression levels of sncRNAs are displayed from yellow (high expression) to blue (low expression). Samples that are pools of RNA from different individuals are indicated as pool.

To explore potential variations in sncRNA transcriptome patterns between fetal and adult brain groups, we conducted a differential expression analysis, applying criteria of |FC| ≥ 2.0, adjusted p-value ≤ 0.05, and focusing on molecules with 100 or more reads in at least one sample. We detected 104 miRNAs, 28 piRNAs, and 70 snoRNAs showing differential expression between the analyzed groups (Supplementary Tables 5–7). The fetal brain transcriptome was characterized by 82 miRNAs, 17 piRNAs, and 70 snoRNAs showing higher expression and 22 miRNAs, 11 piRNAs with lower expression in comparison to the adult brain. These findings are depicted by heatmaps in Fig. 2a–c. Interestingly, the significantly enriched miRNA hsa-mir-29b-3p in the adult brain (71.7-fold) has recently been linked to cognitive function^39^. Concerning piRNAs, the notably abundant piR-hsa-9491 (10.1-fold) and piR-hsa-12487 (5.5-fold) in adults in our study were reported as significantly downregulated in glioblastoma patients^40^.

Thus, considering the limited knowledge about cell type-specific sncRNAs in the human brain, we provide this resource for future studies aiming to understand sncRNA-related molecular mechanisms of brain function and disease.

## Methods

### Sample acquisition

Samples of human total RNA from different brain regions were obtained from BioChain Institute (USA) and TaKaRa Bio Inc. (USA) (Supplementary Table 1). 2 RNA samples of total brain and 22 RNA samples from 16 brain regions, including cerebellum, cerebral cortex, cerebral meninges, corpus callosum, diencephalon, frontal lobe, hippocampus, medulla oblongata, occipital lobe, olfactory region, parietal lobe, pons, postcentral gyrus, precentral gyrus, temporal lobe, and thalamus, composed the adult group. 5 samples of this group were represented by pooled RNA extracted from brain tissues of 5 different individuals. The donors’ ages ranged from 20 to 87 years (median age: 27 years). The fetal group contained 6 RNA samples from the following brain regions: cerebellum, diencephalon, frontal lobe, occipital lobe, parietal lobe, and temporal lobe, along with 1 sample of total brain. The embryos were at 20–40 weeks of development (median: 24 weeks). As a control for germline piRNAs, 3 human testis RNA samples sourced from BioChain Institute (USA) were also included in the study, two of them were pools of 5 and 39 different individuals (Table 1).

### Library preparation, sequencing, and quality control

For small RNA sequencing, 1 µg of total RNA per sample was used as the starting material for preparing indexed libraries using the TruSeq Small RNA Sample Prep Kit according to the manufacturer’s protocol (Illumina, USA). Briefly, 3′ and 5′ adapters were sequentially ligated to small RNAs, and reverse transcribed to generate cDNA. 15 cycles of PCR were performed to amplify and index the samples. PCR products were gel-purified on a 6% polyacrylamide gel, selecting species shorter than 200 nucleotides in length, and were subsequently ethanol-precipitated. Library quality and quantity were verified by analysis on the Agilent Bioanalyzer DNA1000 chip (Agilent Technologies, USA) and Qubit ds DNA HS Assay Kit (Thermo Fisher Scientific, USA). Each library (3 pM) was sequenced for 50 cycles on the HiSeq 2500 platform (Illumina, USA).

For the analysis of expression of piRNA pathway genes, one sample of total adult brain RNA and one sample of testis RNA were subjected to RNA sequencing performed using the TruSeq Stranded Total RNA Sample Prep Kit, starting from 1 µg of total RNA (Illumina, USA). RNA libraries were sequenced at a concentration of 8 pM for 2 × 100 cycles on the HiSeq 2500 instrument (Illumina, USA).

### SmallRNA-seq analysis

The WIND pipeline was used for preprocessing, alignment, and annotation of small RNAs^32^. In brief, a quality control check of the smallRNA-seq data was carried out using the FastQC tool^41^ and adapter removal was performed using Cutadapt^42^. Two methods were used to assess the expression of sncRNAs: one involved aligning reads to a reference genome using STAR^43^ and quantifying the aligned reads using FeatureCounts^44^, the other method estimated transcript level abundance using Salmon^45^, an aligner-free technique. The Salmon and FeatureCounts findings were combined, and only the molecules supported by the two methods were considered. For normalization and differential expression analysis (DE), the DESeq 2 package (v1.42.0) with the DESeqDataSetFromMatrix(), estimateSizeFactors(), and counts() functions was applied^46^. For the assessment of sncRNA expression in biological and technical replicates, correlation matrices were created using corrplot from the R-package. Circos plots were generated using Circos^47^ and heatmaps using the Multi Experiment Viewer software v4.9^48^.

### Real-time quantitative PCR

1 µg of total RNA was used to generate cDNA with the AffinityScript cDNA Synthesis Kit (Agilent Technologies, USA), according to the manufacturer’s instructions. Primer sets specific for piRNA pathway genes were designed using Primer3 and their sequence information is available in Sellitto *et al*.^49^. cDNAs were diluted to a final concentration of 20 ng per reaction. Real-time quantitative PCR was performed in triplicate using Brilliant II SYBR Master Mix (Agilent Technologies, USA) on Mx3005P Instrument (Agilent Technologies, USA). The gene expression levels were normalized against *GAPDH* mRNA.

## Data Records

Sequencing data generated in the study were deposited in the EBI ArrayExpress database (http://www.ebi.ac.uk/arrayexpress) with the following accession number: E-MTAB-13690^50^.

## Technical Validation

### RNA quality

The quality assessment of the RNA samples was performed by measuring the RNA integrity number (RIN) on the Agilent 2100 Bioanalyzer (Agilent Technologies, USA). All RNA samples were of sufficient quality for subsequent library preparation. RIN values ranged from 5.8 to 8.9, with a mean ± SD of 7.37 ± 0.86 (Table 1).

### Sequencing quality

In total, more than 1.4 billion raw reads were acquired, averaging 34.15 ± 6.49 million per sample (Supplementary Table 2). Out of them, a mean of 31.45 ± 6.26 million reads were mapped to the human genome. The sequenced libraries showed an average GC content of 45.81 ± 1.96%. The mean Phred score for individual samples varied from 32.03 to 36.25, indicating high accuracy in base calling during sequencing. The reads with a Phred score of less than 20 were filtered out.

### Reproducibility of technical and biological replicates

In order to check the accuracy of library preparation and sequencing procedures, we sequenced two total brain RNA samples and three testis RNA samples in duplicate or triplicate. Pearson’s correlation coefficients were calculated to assess consistency in sncRNA expression across these replicates. In all cases, correlation values were determined to be 1, indicating complete concordance and repeatability in the technical assessments (Supplementary Fig. 3a).

Similarly, to validate the reproducibility between biological replicates, we correlated sncRNA expression data for the samples related to the same brain region within the adult group (Supplementary Fig. 3b). On average, Pearson’s correlation coefficients were significantly higher for biological replicates (0.96 ± 0.02) than for samples from different brain areas (0.82 ± 0.15).

These results demonstrate consistent technical accuracy and reproducibility of the experimental and biological replicates in our study.

### Evaluation of piRNA pathway gene expression in the human brain

**Fig. 3:**
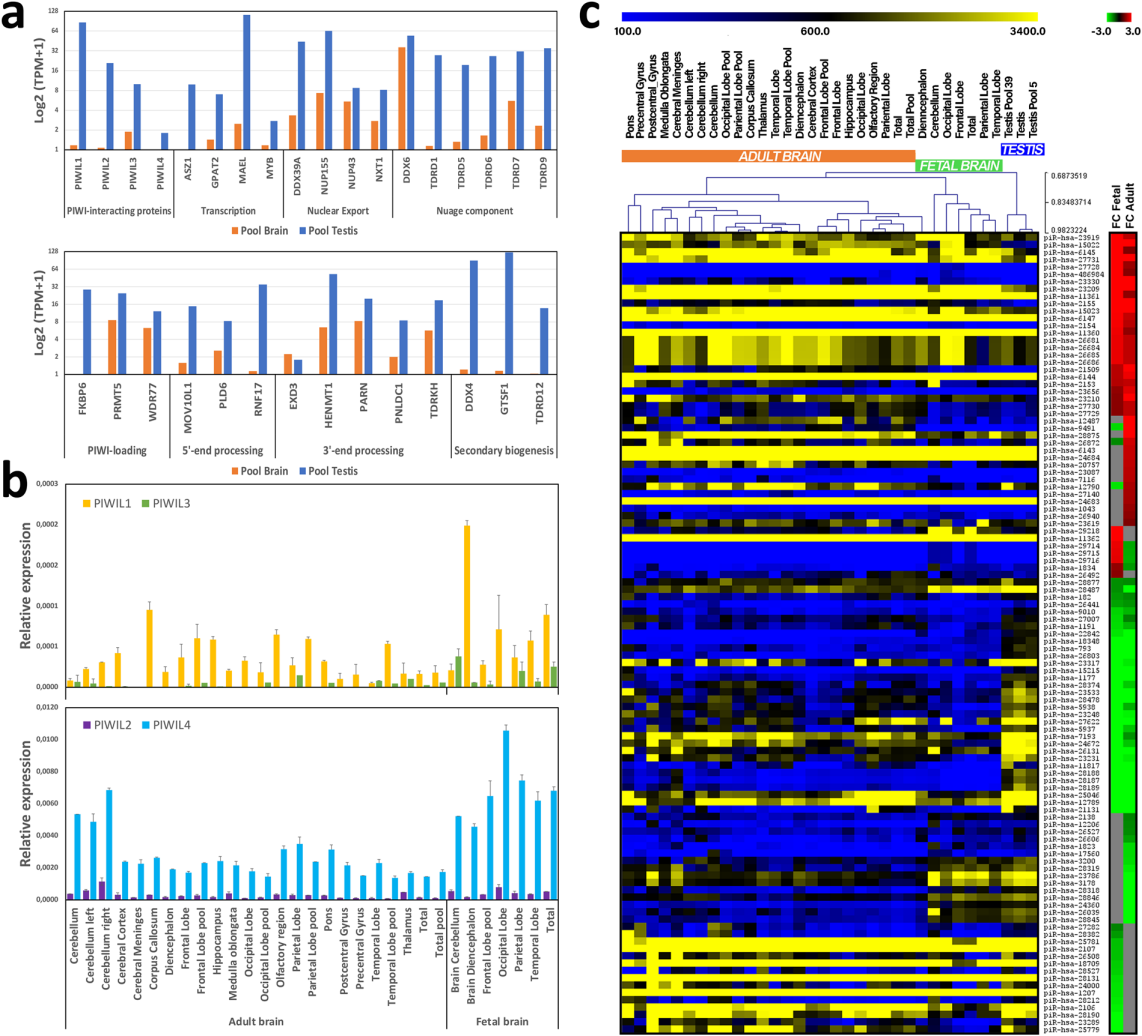
Validation of the PIWI-piRNA pathway activity in the human brain. (**a**) Expression of genes involved in piRNA biogenesis by RNA-Seq analysis of pooled brain and testis samples. The genes were categorized by their respective role in the piRNA pathway. (**b**) Real-time PCR quantification of *PIWIL1*, *PIWIL2*, *PIWIL3*, and *PIWIL4* gene expression in adult and fetal brain samples. (**c**) Heatmaps depicting expression (left) with respect to fold-change (FC) (right) of differentially expressed piRNAs in adult and fetal brain samples compared to the testis group. Only piRNAs with a read count ≥ 100 in at least one sample with |FC| ≥ 1.5 and adjusted p-value ≤ 0.05 were considered. Expression levels are displayed from yellow (high expression) to blue (low expression) while FCs are displayed from green (under-expressed) to red (over-expressed). Samples that are pools of RNA from different individuals are indicated as pool.

To validate the presence of piRNAs in the human brain, we analyzed piRNA pathway activity and functionality. We used RNA-sequencing data to conduct an expression screening of genes known to influence their biogenesis and mechanism of action, comparing one sample of total adult brain RNA alongside a testis RNA sample. Our findings revealed a noteworthy expression of genes encoding for piRNA biogenesis factors in the brain sample, albeit at a lower level compared to the more pronounced expression observed in the testis sample (Fig. 3a). The analyzed piRNA maturation process factors included PIWI-interacting proteins, molecules involved in piRNA transcription, nuclear export, nuage component formation, PIWI-loading, 5′- and 3′-end processing, and secondary biogenesis. Compared to the testis sample, the brain RNA sample exhibited an absence of *FKBP6* expression and almost undetectable expression levels of *PIWIL3*, *ASZ1*, and *GTSF1*.

Then, we assessed the mRNA levels of *PIWI* genes in both adult and fetal brain RNA groups of samples using real-time PCR. The results showed a higher expression of *PIWIL2* and *PIWIL4* genes in contrast to *PIWIL1* and *PIWIL3* (Fig. 3b). Notably, all *PIWIL*-transcripts demonstrated greater abundance in fetal RNA samples than in adult ones. In detail, the mean ± SD values of relative expression for fetal and adult groups were as follows: 7.19E-5 ± 6.11E-5 and 3.34E-5 ± 2.26E-5 for *PIWIL1* (p = 0.037, Wilcoxon rank-sum test), 4.55E-4 ± 1.93E-4 and 2.87E-4 ± 2.25E-4 for *PIWIL2* (p = 0.014), 1.44E-5 ± 1.39E-5 and 0.48E-5 ± 0.39E-5 for *PIWIL3* (p = 0.13), and 6.76E-3 ± 1.94E-3 and 2.6E-3 ± 1.35E-3 for *PIWIL4* (p < 0.001).

### PiRNA brain signature

In order to validate piRNA-specific expression in brain tissues, we also assessed their enrichment in brain tissues against testis samples. We performed a differential expression analysis, considering |FC| ≥ 1.5, adjusted p-value ≤ 0.05, and a minimum read count of 100 in at least one sample (Fig. 3c, Supplementary Table 8). Overall, we identified 109 differentially expressed transcripts. Among these, 25 piRNAs exhibited higher expression, while 32 displayed lower expression in both the fetal and adult groups compared to the testis group. 12 piRNAs were expressed at significantly higher levels and 15 at lower levels solely in the adult group when compared to the testis group. 3 piRNAs exhibited higher expression, and 15 showed lower expression in the fetal group as opposed to the testis group. Finally, 4 piRNAs were expressed at higher levels in fetal and at lower levels in adult samples in contrast to the testis group, while 3 piRNAs displayed the opposite pattern, expressed at lower levels in fetal and at higher levels in adult samples when compared to the testis group.

Taken together, these results strongly suggest that the PIWI-piRNA pathway is active in the human brain.

### Supplementary information

Supplementary Figures

Supplementary Tables

## Data Availability

No custom code was used to generate or process the data described in this manuscript.
