# Supplementary material for: Small non-coding RNA transcriptomic profiling in adult and fetal human brain
Source: Sci Data. 2024 Jul 12;11:767. doi: 10.1038/s41597-024-03604-6 (PMC11245507; doi:10.1038/s41597-024-03604-6)
Supplement: Supplementary file 1 — Supplementary Figures [file 41597_2024_3604_MOESM1_ESM.pdf]

Supplementary Information

Supplementary Fig. 1

Supplementary Fig. 2

Supplementary Fig. 3

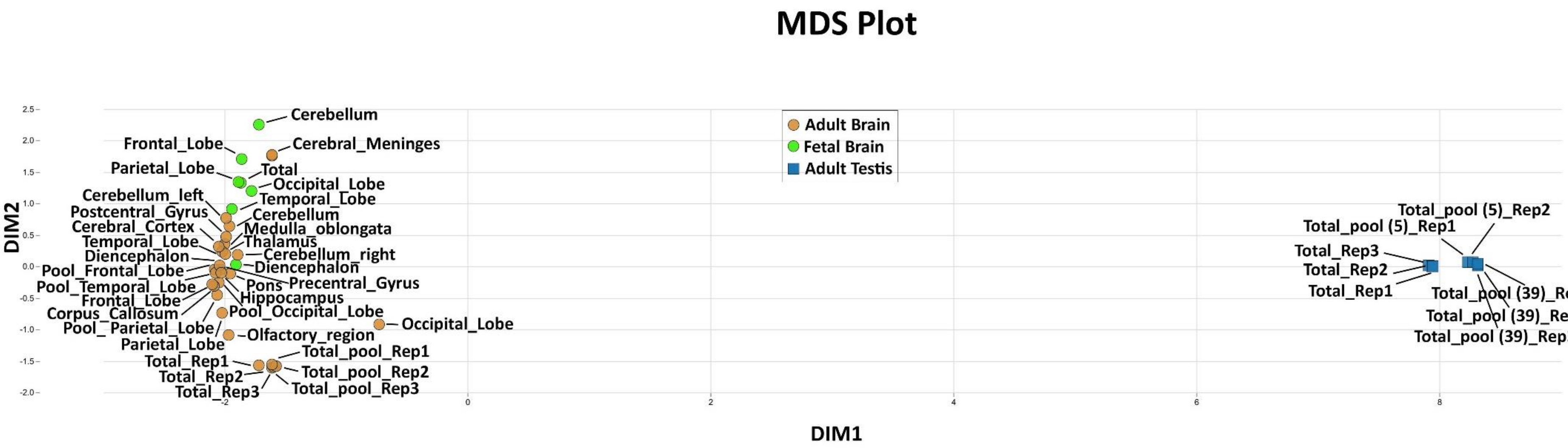

Supplementary Fig. 1. Multidimensional Scaling (MDS) plot of sncRNA profiles in brain and testis tissues. The MDS plot reveals that samples cluster according to tissue type, with further sub-clustering observed based on developmental stage within the brain group. Samples that are pools of RNA from different individuals are indicated as pool.

A

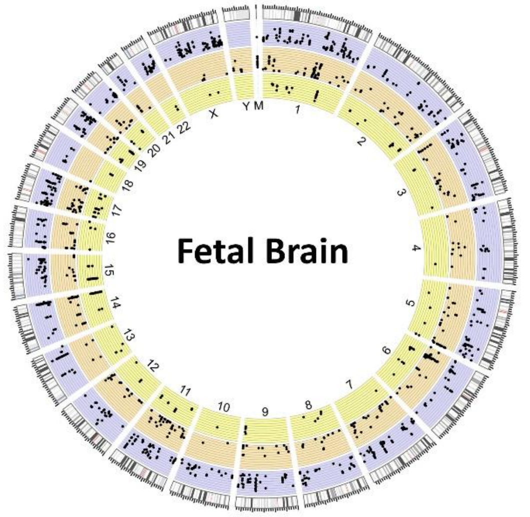

B

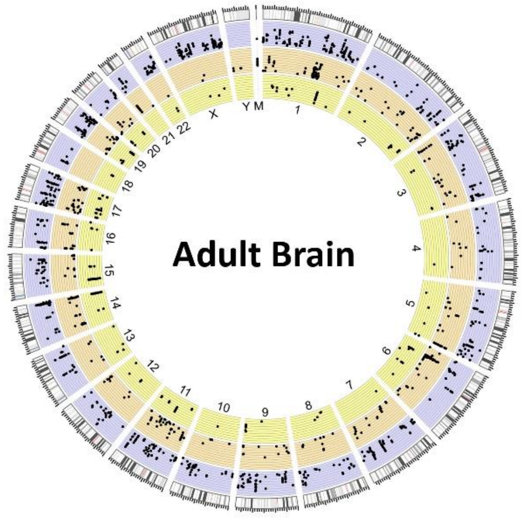

C

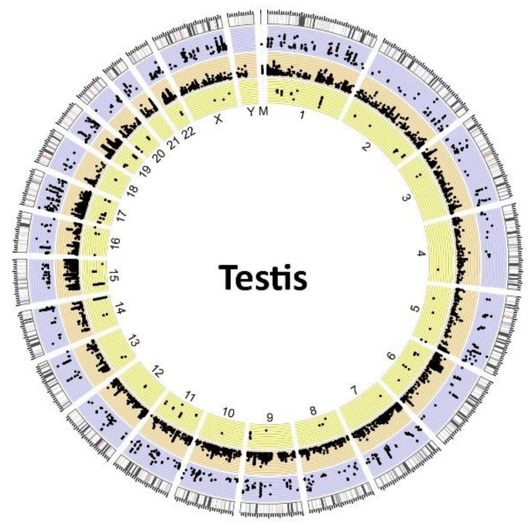

Supplementary Fig. 2. Genome-wide distribution of sncRNA species in human brain and testis. Circos plots illustrating genomic positions and mean log2 (count+1) expression levels of miRNAs (blue), piRNAs (orange), and snoRNAs (yellow) detected (reads ≥10) in the fetal brain (a), adult brain (b) and testis (c). M: mitochondrial genome.

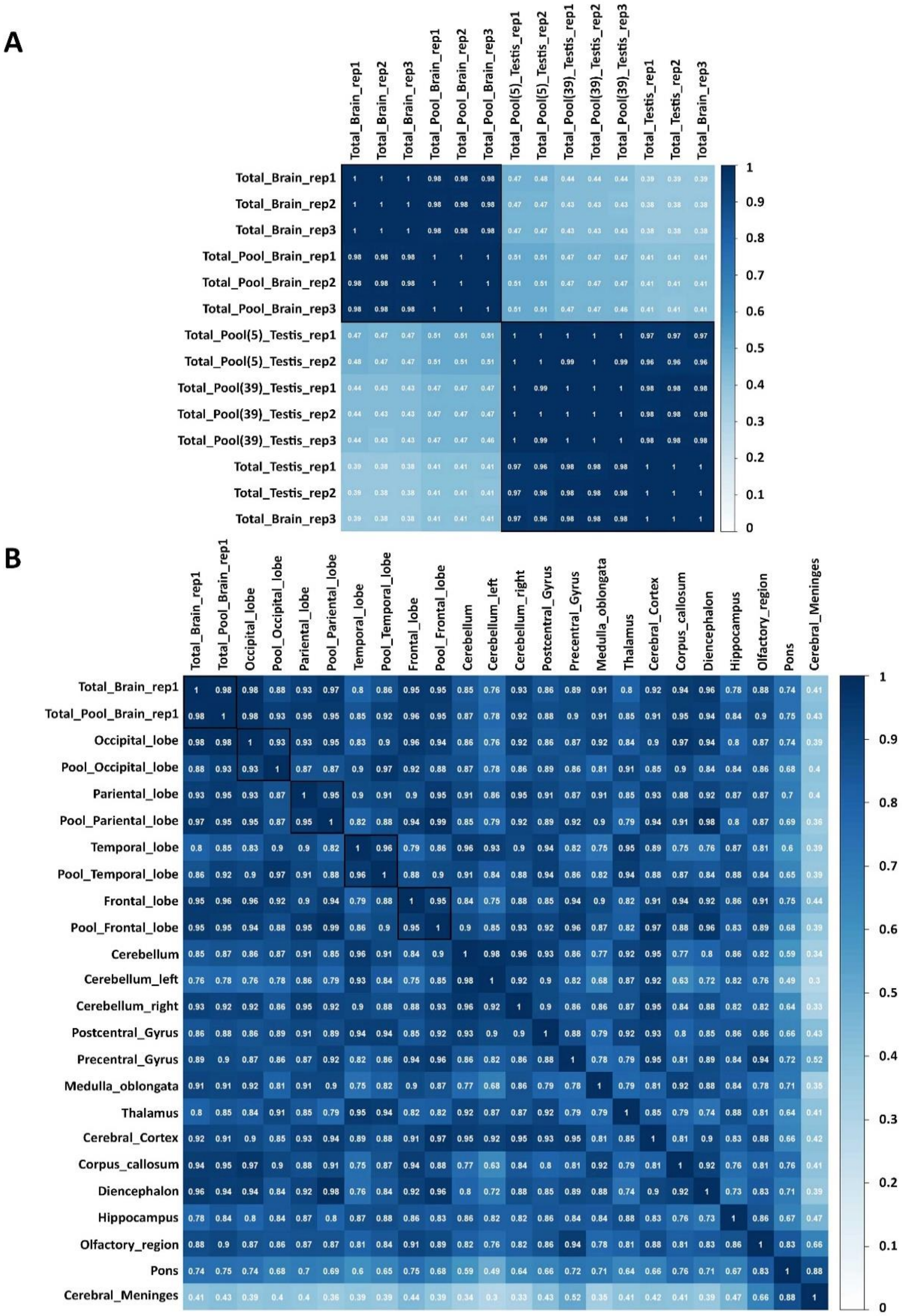

Supplementary Fig. 3. Reproducibility of technical and biological replicates. (a) Correlation matrix showing the Pearson correlation coefficients (r) between sncRNA expression levels in technical replicates. (b) Pairwise Pearson correlation coefficients among biological replicates and across samples from different adult brain regions; the darker the color, the more correlated the samples. Samples that are pools of RNA from different individuals are indicated as pool.
